# Quinine

**Published:** 1848-12

**Authors:** Wm. Fletcher Holmes

**Affiliations:** Newberry, S. C.


### RECORD OF MEDICAL SCIENCE.

*Quinine.* By Wm. Fletcher Holmes, M. D., of Newberry, S. C. —There are various conflicting opinions in relation to the action of Quinine upon the animal economy; some authors advocating its sedative, and others its excitant properties: whilst perhaps a majority regard it as either stimulant or sedative, according to the dose. In large doses, experience has led me to look upon it as a sedative to the vascular, but an excitant to the nervous system, producing a high degree of nervous erethism, a peculiar irritability of the nerves, which supply the organs of hearing and vision, and a vertiginous sensation described by patients as extremely distressing. This tendency which quinine possesses, of augmenting, and indeed originating determination to the brain, has led to its exclusion in fevers accompanied with cerebral congestion, or inflammation, or nervous excitability. For some time past, physicians have been on the *alerte* to discover some agent which would neutralize this peculiar property of quinine without impairing its febrifuge powers. The west and south-west have exultingly proclaimed "Eureka," and hold up to us morphine as the long sought desideratum. But impartial observation will convince any eclectic practitioner that any preparation of opium will enhance to an alarming extent the prevailing determination to the brain. Ever since I took my degree, this subject has been a matter of inquiry and experiment with me, and I have come to the decided conclusion that *Digitalis*, modifies the action of quinine, in this particular, to a more considerable extent than any other agent. I have administered quinine in large doses, in combination with this medicine, to young children, whose nervous systems were very mobile and impressible, with the happiest effects, and without producing any of those unpleasant symptoms, commonly attributable to the free exhibition of this potent febrifuge. To delicate females, whose constitutional aversion to this medicine amounted almost to idiosyncrasy, it has been given with a few drops of the tincture digitalis, with the most pleasant results. In bilious remittent, I do not hesitate to exhibit quinine, as mentioned above, even when the exacerbation is at its height, and the consequences are often seen in an abundant flow of the cutaneous transpiration, a prolonged remission, and in many instances a complete apyrexia.

I regard quinine as possessing high antiseptic properties. In malignant and protracted fevers, where it is desired to make a mercurial impression upon the system, in combination with calomel, it (quinine) assists in the development of ptyalism, and at the same time modifies the well known tendency of that powerful alterative to produce ulceration of the gums, sloughing of the cheeks, etc. In typhous and typhoid fevers, it has seemed to me to correct the indisputable proclivity to putrescency of the fluids, as indicated by petechiæ, and the prompt disposition to gangrene of vesicated surfaces. In the dothenenterite of typhoid fever, in combination with calomel and the nitrat. argent., it has been given with the happiest effects ; and in the " congestive chills" which have latterly sprung up into such gloomy notoriety among us, it is the sheet anchor of the physician's hopes, and sooth to say, it rarely fails us in the hour of trial. Upon its abundant exhibition alone must the practitioner base his expectation of success, and its rapid and powerful action often transcends our most sanguine anticipations.

The attention of the profession has hitherto been solely directed to the action of quinine as a febrifuge, overlooking what I consider of paramount importance, viz: its special affinity for the nervous system, and its peculiar adaptation to the relief of neuropathic affections of long standing.

In a protracted case of neuralgic dysmenorrhœa, accompanied with intense cephalalgia, which resisted cups and vesication, in which the most powerful alteratives, such as mercury, guaiac., iodine, and the arsenical solution had been vainly tried, I administered quinine combined with carb. ferri and belladonna, with decided effect.

This may seem inconsistent with my belief in its stimulant property, and I can account for its efficacy in this instance upon no other grounds than its obvious analogy with the beneficial exhibition of stimulants in the chronic phlegmasiæ. In the treatment of epilepsy, chorea St. Viti, I am disposed to look for important results from the use of quinine.—*Charleston Med. Jour.*

